# Pulmonary Appoplexy—Consequent on Ulceration of the “Os Aud-Cervix Uteri”

**Published:** 1867-02

**Authors:** E. F. Knott

**Affiliations:** Atlanta, Ga.


NEW SERIES.

Vol. VIII. FEBRUARY, 1867. No. 12.

### ORIGINAL COMMUNICATIONS.

### ARTICLE I.

*Pulmonary Appoplexy—Consequent on Ulceration of the "Os aud-cervix uteri."* By E. F. Knott, M. D., of Atlanta, Ga.

On the 5th of September, 1866, I was called to see Adeline, a mulatto, aged 17. I found her laboring under the following symptoms: A profuse flow of bloody, frothy serum, from the mouth and nose; dullness over the entire surface of the right lung, with some dullness over the left lung; complains of violent pain in the hypochrondiac regions; great oppression about the precordia, with more or less pain seated in the supraorbital regions; pulse full, with evidence of local determination; repeated convulsions; the patient, during these convulsive attacks, resting on the heels and occiput, while, at the same time, she would grasp with her hands the integument over the precordiac, with such firmness and tenacity as to render it exceedingly difficult to remove them. Venesection, to the amount of twenty-four ounces, counter-irritation, over the chest and along the spine, followed by plumbi acetas et opii every three hours.

Sept. 6th.—Symptoms about the same; hemorrhage persisting. The bowels having been constipated for several days, ordered ol. recini f ℥ i., terebinthina f ʒ i.

Sept. 7th.—Medicine has acted freely. Hemorrhage still continues, but less freely. Ordered the plumbi acetas and opii continued as before. At my visit in the afternoon found no alteratian in the patient's condition. Treatment continued.

Sept. 8th.—Hemorrhage continues. No abatement of the other symptoms. Ordered comp. cathartic pills (U. S. P.), one every hour until they operate. Saw the patient again in the afternoon. The pills not acting, ordered olei ricini f℥ i., S. ft. hunstis.

Sept. 9th.—Medicine has acted freely. But little alteration in the symptoms. Ordered turpentine emulsion. Evening visit: finding evidences of local determination more prominent, practiced venesection to the amount of sixteen ounces.

Sept. 10th.—But slight amelioration of symptoms. Cupped freely, over the chest, and along the whole extent of the spine. After this, applied sinapism from sacrum to nape of neck, with a view of overcoming the spasmodic attacks, which have been persistent during her illness.

Sept. 11th.—Found the patient in the following condition: Resting on her back, inferior extremities extended, jaws firmly locked, so much so as to resist all force employed to separate them, short of doing violence to the parts concerned. Patient perfectly conscious of all that occurs about her. It being impracticable to administer anything by the mouth, ordered ol. ricini f℥ ii., ol. terebinth f ʒ ii., tepid water f℥ xvi., one half to be used as an enema, to be followed in the course of two hours by the other half, provided the first does not move the bowels. Counter irritation to the spine and chest to be kept up.

Sept. 12th.—Condition of the patient the same. Requested my son, Dr. J. J. Knott to see the case with me. He suggested the probability of some organic affection of the womb. A vaginal examination, *per speculi*, revealed ulceration of the "os aud cervix uteri." Cauterized the parts with caustic, and ordered bromide potassium ʒ v., ext. lactucæ ʒ i., aqua f ℥ xvi., fiat sol. Half of this was administered per rectum. About two o'clock in the afternoon, the other half was administered in the same manner. Saw the patient again, at 9 o'clock, P. M., of same day: found decided improvement of all the symptoms; entire absence of all tetanic symptoms. Hemorrhage very slight.

Sept. 13th.—Found the patient comfortable. Ordered bromide potassium ʒ ii., ext. lactucæ ʒ ii., aqua pura f ℥ ii., ft. sol., S., teaspoonful every other hour.

Sept. 14th.—Patient still improving. Treatment continued.

Sept. 15th.—Patient convalescent. Bromide potassium continued at intervals of six hours.

Sept. 16th.—Patient dismissed, with orders to continue the menicine as last directed.

Remarks.—Up to the present time the patient has remained well, not having had an attack since.

Some twelve months previous to the patient's last illness she had been confined with her first child. Some three or four days after her confinement, she was attacked with puerperal fever, which confined her to her bed for three months. After getting up from this attack, she experienced frequent slight attacks of hemoptysis, though they were not of sufficient import, as she thought, to call in medical aid. These attacks, though, were not complicated with any spasmodic symptoms. During this time she was irregular in her catamenia, which irregularity, together with her other affection, must have depended upon the organic lesion of the womb, as all the symptoms yielded readily to the local and general treatment adopted in the latter stage of her illness.

The foregoing detail is given in consideration of the peculiarity of the case, and in view of still further illustrating the effect following the use of bromide of potassium, when employed in cases where the womb may be regarded as the seat of the disease. The querry here presents itself to my mind, Why may not this remedy be successfully employed in the treatment of tetanus? We know it to be a powerful nervous sedative.

